# Tumor flare reaction with brentuximab-vedotin in mycosis fungoides

**DOI:** 10.1016/j.jdcr.2026.01.051

**Published:** 2026-02-06

**Authors:** Rishabh Lohray, Seda Purnak, Samruddhi Arkatkar, Madeleine Duvic, Victor Prieto, Carlos Torres-Cabala, Auris Huen

**Affiliations:** a Department of Dermatology, Baylor College of Medicine, Houston, Texas; b Department of Dermatology, University of Texas MD Anderson Cancer Center, Houston, Texas; c Department of Pathology, University of Texas MD Anderson Cancer Center, Houston, Texas

**Keywords:** brentuximab vedotin, mycosis fungoides, tumor flare

## Introduction

Brentuximab vedotin (BV) is an anti-CD30 antibody conjugated to the microtubule assembly inhibitor monomethyl auristatin E. It is Food & Drug Administration-approved for the treatment of CD30+ mycosis fungoides (MF) with one of the highest overall response rates (65%), especially in treating tumors and nodal disease. While peripheral neuropathy is the most frequently reported adverse effect, tumor flare reaction (TFR) has rarely been documented. This case report presents a patient with TFR with the intent of educating providers on this phenomenon and preventing unnecessary therapy cessation.

## Case report

**Fig 1:**
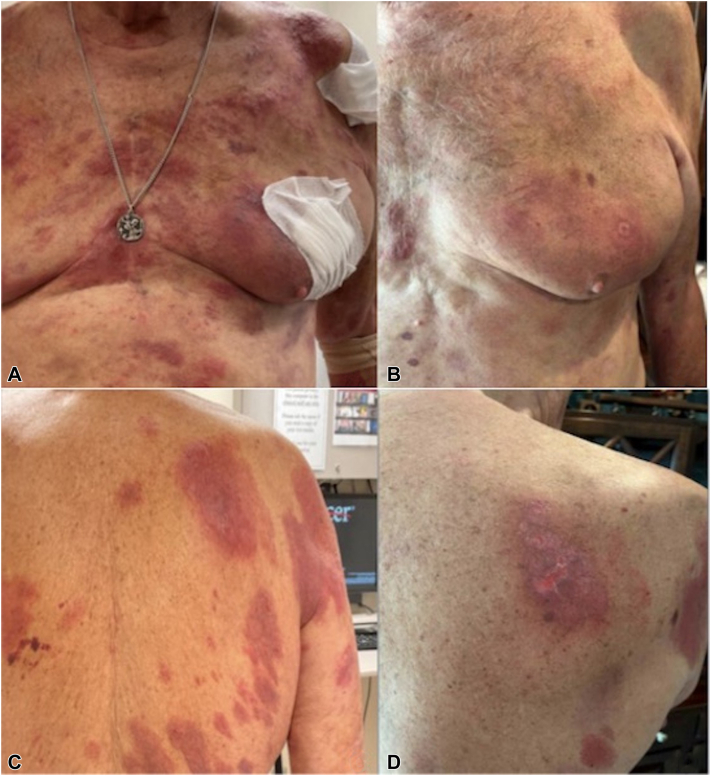
**A** and **C,** Tumor flare reaction with extensive erythematous plaques and tumor on chest and back after initiation of brentuximab vedotin (cycle 1, day 14) that (**B** and **D**) markedly improved following continued therapy with BV at 1.2/kg/mg (cycle 2, day 21). *BV*, Brentuximab vedotin.

An 80-year-old White male with a history of stage IIB folliculotropic MF with CD30+ large cell transformation and gamma/delta phenotype presented with skin-limited disease without nodal, visceral, or blood disease. He had failed multiple therapies, including topical nitrogen mustard, topical corticosteroids, topical tazarotene, topical bexarotene, acitretin, and interferon. He plateaued in response to narrow-band UV-B and failed to achieve a durable response to multiple treatments of low-dose local electron beam and total skin electron beam therapy. Intravenous BV was initiated at a dose of 1.8 mg/kg given every 3 weeks. The patient's disease had been resistant to treatment but stable for months before BV initiation (pre-BV body surface area [BSA], modified severity-weighted assessment tool [mSWAT]: 20.4%, 28.9). BV was given as monotherapy, and no systemic steroids were given. Two weeks after the first dose, the patient developed significant worsening of his skin disease (BSA, mSWAT: 50%, 90) with worsening patches and plaques on his face, trunk, extremities, tumor on chest, and blisters on feet (Fig 1, *A* and *C*).

A biopsy of one of the new lesions revealed a lymphoid infiltrate with focally large, CD4–/CD8–, CD30–, T-cell receptor delta positive, epidermotropic cells consistent with MF, with scattered cytotoxic CD8+ T-cells. Wound cultures were negative. BV was discontinued due to presumed progression, and total skin electron beam therapy was planned. However, 2 weeks later, most of his new lesions had resolved, showing a response to BV. Hence, BV was restarted at 1.2 mg/kg every 3 weeks, and the patient responded well with clinical improvement and no flare (BSA/mSWAT at last visit: 4%, 4; Fig 1, *B* and *D*). A dose reduction was done to avoid another tumor flare. The cumulative BV dose at the last visit was 3.1 mg/kg.

## Discussion

TFR is classically associated with immune-related therapies such as immune checkpoint inhibitors, and in the case of lymphomas, can present as splenomegaly and lymphadenopathy. Histopathologically, it is often characterized by an infiltration of natural killer or cytotoxic cells.^1^ TFR specifically linked to BV use in MF has rarely been documented. Zhu et al report a case of TFR in a patient with Hodgkin's lymphoma who was on a BV + tislelizumab combination therapy, though tislelizumab, an immune checkpoint inhibitor, alone can also cause TFR.^2^ Because the lymph node biopsy was delayed, neither reactive cells nor tumor cell infiltration were identified, despite clinical evidence of lymphadenopathy.^2^ In a study of 58 patients with systemic anaplastic large cell lymphoma treated with BV, 4 experienced TFR characterized by tender lymphadenopathy and overlying erythema; all regressed radiographically.^3^

Our patient’s flare was indistinguishable from progression, even with biopsy and cultures. Observation ultimately revealed regression, allowing therapy continuation. This parallels a prior patient at our institution, who developed paradoxical worsening of MF lesions after the first BV dose in a phase II clinical trial by Duvic et al.^4^ Four days after BV initiation at 1.8 mg/kg, the patient noticed worsening maceration, erosions, swelling, erythema, and new tumor formation (BSA >90%). A biopsy was not performed at the time of the flare, and the patient noticed improvement in lesions by the time of his second dose of BV (BSA: 21.7%). Hence, the initial increase in BSA was presumed to be a TFR, and the patient received a total of 19 infusions at the same dose over the next year with near complete resolution of his lesions. Six years after BV cessation, the patient continued to have excellent disease control and durable remission (BSA: 4.5%). Besides this case, no other case of BV linked to TFR has been reported in MF.

The mechanism by which BV induces TFR is unclear. However, some patients may be susceptible to a flare due to prior immune modulation, such as our patient who had previously received pegylated interferon alfa-2a. TFR during BV treatment of MF has clinical relevance because a robust increase in BSA involvement after the first BV dose could prompt a premature diagnosis of progression. Although an appropriate work-up with a detailed clinical history, physical exam, and skin biopsy may help in diagnosing TFR, this did not clarify the distinction between TFR and progression in both of our cases. Observation over 1-2 weeks appears to be the most practical approach to allow for differentiation before therapy discontinuation.

This case highlights BV-associated TFR as a rare but clinically significant phenomenon in MF. Physicians should maintain a high index of suspicion for TFR when confronted with abrupt disease worsening after initial BV doses, particularly in patients with prior immunomodulatory therapies. Careful counseling regarding the risks and benefits of discontinuation versus continued treatment with palliative therapy is essential.

## Conflicts of interest

None disclosed.
